# Heavy Metals in Soil and Crops of an Intensively Farmed Area: A Case Study in Yucheng City, Shandong Province, China

**DOI:** 10.3390/ijerph7020395

**Published:** 2010-02-01

**Authors:** Lin Jia, Wuyi Wang, Yonghua Li, Linsheng Yang

**Affiliations:** 1 Institute of Geographic Sciences and Natural Resources, Chinese Academy of Sciences, 11A Datun Road, Beijing 100101, China; E-Mails: jial.07b@igsnrr.ac.cn (L.J.); yhli@igsnrr.ac.cn (Y.L.); yangls@igsnrr.ac.cn (L.Y.); 2 Graduate School of the Chinese Academy of Sciences, Beijing 10049, China

**Keywords:** heavy metals, multivariate statistics, agricultural activities, Yucheng City

## Abstract

Yucheng City is located in northwestern Shandong Province, China, and is situated on the Huang-Huai-Hai Plain, the largest alluvial plain in China. In this study, 86 surface soil samples were collected in Yucheng City and analyzed for cation exchange capacity (CEC), soil organic matter (SOM), pH, available phosphorus (avail. P), phosphorus (P), aluminum (Al), and iron (Fe). These soils were also analyzed for ‘total’ chromium (Cr), nickel (Ni), copper (Cu), zinc (Zn), arsenic (As), mercury (Hg), cadmium (Cd), and lead (Pb), together with 92 wheat samples and 37 corn samples. There was no obvious heavy metal contamination in the soil and irrigation water. But the long-term accumulation of heavy metals in soil has lead to an increase of Ni, As, Hg and Pb concentrations in some of wheat and corn samples and Cd in wheat samples. Because of the numerous sources of soil heavy metals and the lower level of heavy metal in irrigation water, there is no significant relation between soil heavy metal concentrations and irrigation water concentrations. Cr, Ni were mainly from the indigenous clay minerals according to multivariate analysis. Little contribution to soil heavy metal contents from agricultural fertilizer use was found and the local anomalies of As, Cd, Hg, Pb in wheat and corn grain are attributed to the interactive effects of irrigation and fertilizer used. Aerial Hg, however may also be the source of Hg for soil, wheat and corn.

## 1. Introduction

China is currently experiencing rapid development and extensive social and economic structure changes. The industrial sector has contributed much to economic development in the last three decades, but agriculture has been the main base for China’s economy, especially in the intensively farmed areas. The rapid development of industry, however, has resulted in a lot of waste going into drains, which contaminates rivers, streams, and local channels. Streams, rivers, and channels are the primary sources of water for irrigation [1]. Irrigation with water polluted with heavy metals may also lead to soil and plant pollution [2]. In intensively farmed areas, agricultural development is also accompanied by the use of large amounts of pesticides and organic and inorganic fertilizer inputs. Increased use of chemical fertilizers and livestock and poultry manure can also lead to an increase in heavy metals such as Cd, Pb, Cu, and Zn in soils and plants [3].

The Huang-Huai-Hai Plain (North China Plain) is the largest alluvial plain in China, and supports an important agricultural industry that accounts for ~23% of national food production [4]. Since the 1990s the Huang-Huai-Hai Plain has been developed into an important production base that serves as a model for agricultural development in North China [5]. The agricultural intensification level is increasing with the growth of the regional population and improvement and comprehensive management of saline and alkali soils since the 1980s [6]. The agricultural production has shifted from drought and flood disasters to drought disasters. Reusing straw and stalks of crops and applying more organic fertilizers are considered the main measures to improve the saline and alkali soils in the Huang-Huai-Hai Plain [6]. Increasing agricultural intensification, such as the increasing inputs of agricultural machinery, fertilizer, pesticide, and irrigation water, and climatic and environmental changes may be posing threats to the local soil and crop quality. Yucheng City in Shandong Province, China, typifies the Huang-Huai-Hai Plain’s landscape, water, and soil resources. The nature and extent of agricultural development and industrialization are also typical of the wider Huang-Huai-Hai Plain [7]. Investigations into heavy metals in Yucheng’s agricultural areas will therefore be applicable to environmental protection and agricultural sustainability across the the Huang-Huai-Hai Plain and China as a whole.

Heavy metals are ubiquitous in the environment, either naturally or anthropogenically. Pollution from industrial emissions, wastewater irrigation, and fertilizer waste are the main sources of heavy metal pollution in China and have been intensively studied [8]. However, the lack of investigation into contaminants in agricultural soils and crops derived from different types of agricultural activities in intensive agriculture areas makes it difficult to identify potential problems from certain agricultural practices in China. Thus, it is imperative to identify the extent and severity of heavy metal pollution in soils and crops from different agricultural activities in the intensively farmed area in China. This study aims at this by using principal component analysis (PCA) and correlation analysis of soil properties and heavy metal concentrations. We characterized the heavy metal concentrations in soil and crops and analyzed the Bioconcentration Factor (BCF) of individual heavy metal. Simultaneously, the present study is focused on the probable sources of heavy metals in soil and crop samples as irrigation, certain fertilizer types, and other factors through multivariate analysis in Yucheng City. These aspects have not previously been studied in Yucheng City and have an extreme importance in evaluating the environmental impacts on agriculture.

## 2. Methods and Materials

### 2.1. Study Area

**Figure 1.**
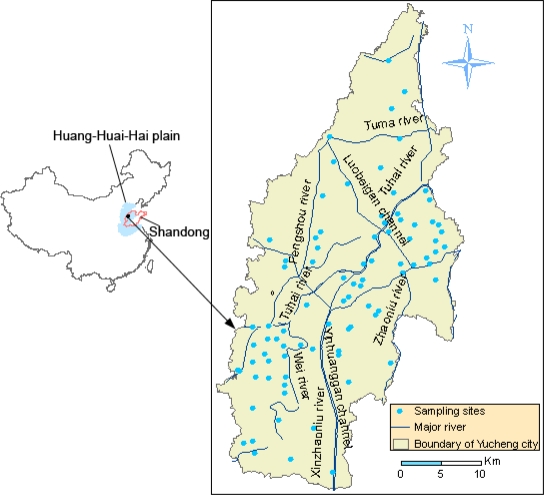
Soil sampling points in Yucheng City, Shandong Province, China.

Yucheng City is situated in the Bohai Economic Rim and is one of the regional central cities in northwestern Shandong Province, China. The total area of is 990 km^2^, and the population is 500 thousand. It is also located on the Yellow River alluvial plain (Figure 1). The area lies within the warm and semi-humid monsoon climate zone, with an annual average temperature of 13.1 °C and an average annual rainfall of 593.2 mm. The soil was formed on the alluvium of the Yellow River. The main soil types are fluvo-aquic soil and solonchak [9], referred to the Chinese Soil Taxonomy system, Chinese Reference Base for Soil Resources [10] recognized by the international scientific community [11–14].The fluvo-aquic soil has the following sub-categories: salinized fluvo-aquic soil, cinamon fluvo-aquic soil, wet fluvo-aquic soil and the sub-categories of solonchak is mainly meadow solochak in Yucheng City [10,15]. The soil has a mild to moderate degree of salinity.

The city is heavily industrialized and intensively cultivated (52.37 thousand ha of agricultural topsoil). The industrial and mining land use contributed most to total increment of the construction land area and achieved 18.84% of total land use in Yucheng in 2005 [16]. There are six major industry clusters in Yucheng City: functional sugar industry; equipment manufacturing; semi-worsted cashmere industry; wood-based panel industry; green food industry and bio-pharmaceutical industry, which drain sewage into the surface water. There are three sources for farmland irrigation: local rivers, groundwater, and the Yellow River channels. The major rivers of Yucheng City include the Tuhai River and its branches, the Zhaoniu River and the Wei River (Figure 1). The Tuhai River runs through the city and receives some untreated wastewater from Yucheng City, while most wastewater and domestic sewage drains into the Luobeigan Channel and the Zhaoniu River. Farmers here depend on chemical and organic fertilizer to improve grain production, and especially in the developed livestock breeding area, livestock and poultry manure were almost thrown to the local soils. Yucheng City’s grain production is relatively high, reaching 15 t ha^−1^ due to high agrochemical inputs and irrigation, which is typical on the intensively cropped agricultural areas on the Huang-Huai-Hai plain.

### 2.2. Sampling and Chemical Analysis

Soil samples (86) were collected from different locations in June, 2008, based on the irrigation water and fertilizer used in the study area just before wheat harvest (Figure 1). Before sampling, the typical areas were investigated and oriented according to different agricultural activities including irrigation types and fertilizer used in Yucheng City, respectively. Each soil sample was taken from the plough layer (0–20 cm) at specific typical agricultural area and is defined as a composite one from three samples with a 10 m distance to ensure that the resulting sample better represents the condition of each area. Paired soil and wheat grain were sampled, and wheat samples were mixed together according to the soil samples. Where available, a corn sample was collected in close proximity to each core later in September, 2008. 47, 17, 22 soil samples, 51, 19, 22 wheat samples and 15, 11, 11 corn samples were obtained in the agricultural areas irrigated with local rivers, ground water and the Yellow River, respectively. In the typical areas from different sources of irrigation water, different source of irrigation water was sampled and confirmed on the spot. 80, 6 soil samples, 85, 7 wheat samples and 30, 7 corn samples were got from the area fertilized with chemical manure and organic manure, respectively.

The soils were air dried at ambient temperature and crushed to pass a 2 mm stainless steel sieve. Portions of soil samples (about 50g) were ground to pass a 0.149-mm sieve, then stored in plastic bags prior to chemical analysis. All samples of wheat and corn were washed with deionized water to remove soil particles or dusts adhering to the grain surface. All wheat and corn samples were oven-dried at 60 °C for 24 hours, then ground in a stainless steel mill to a fine powder and stored in plastic bags for further chemical analysis.

The soils were analyzed for the following properties: pH, cation exchange capacity (CEC), soil organic matter (SOM), available P, Al, Fe, Cr, Ni, Cu, Zn, As, Hg, Cd, and Pb, and total P. Soil pH was determined by a pH meter with a soil/water ratio of 1: 2.5 [17]. Available P was extracted by shaking 1.5 g of soil for 30 minutes at 20 °C in 100 mL of a 42% NaHCO_3_ solution at pH 8.5 according to Olsen *et al.* [18]. The organic content in the soil was determined by the potassium bichromate [19]. Cation exchange capacity was determined using NH_4_OAc at pH 7.0, the leaching method of the Soil Survey Staff, Land Development Department (LDD, 1998). After digesting with a mixture of nitric acid (HNO_3_), perchloric acid (HClO_4_) and hydrofluoric acid (HF) [20], total Al, Fe, Cr, Zn, and P were determined by Inductively Coupled Plasma Optical Emission Spectrometer (ICP-OES PE5300DV); As and Hg were determined by Atomic Fluorescence Spectrometer (AFS-830a); and Cd, Cu, Ni, and Pb were determined by an Inductively Coupled Plasma Source Mass Spectrometer (ICP- MS X2).The wheat and corn samples were analyzed for the heavy metals Cr, Ni, Cu, Zn, As, Hg, Cd, and Pb. After digesting with a mixture of nitric acid (HNO_3_) and perchloric acid (HClO_4_) [21], Cu, and, Zn were determined by an Inductively Coupled Plasma Optical Emission Spectrometer (ICP-OES PE5300DV); Cr, Ni, As, Hg, Cd, and Pb were determined by an Inductively Coupled Plasma Source Mass Spectrometer (ICP-MS X2).

For analytical quality control, two certified reference soils and two reference plant materials were digested in a similar manner to the soil, wheat, and corn samples for quality control and to monitor any instrument variability. The reference soils included GBW 07405 (GSS-5) and GBW 07408 (GSS-8) (China National Center for Standard Materials). The certified plant materials were wheat [GBW 08503, GBW 07604(GSV-3)], and corn [GBW 10012, GBW 07604 (GSV-3)]. Accuracy of the analytical method was given as percent recoveries for each of the elements. The results are reported in Table 1. In every analytical batch, 10% samples of each were analyzed repeatedly to ensure the precision and accuracy of analysis. Internal reference standard materials and reagent blanks were also used in the analysis process to ensure the precision.

### 2.3. Statistical Analysis

Statistical analysis and PCA were performed using SPSS 16.0 and Statistica 6.0. Assessment of the normal distribution of the analytical data was carried out through 1-sample K-S test. Non-normal data were log-transformed to improve normal distribution and to reduce the influence of high analytical data. Correlation (r) and analysis of variance (ANOVA) were performed on log-transformed soil analytical data.

Correlation analysis and PCA, based on the correlation matrix, were conducted for the soil chemical data set. The aim of using PCA was to ascertain any patterns in the soil samples in relation to these chemical characteristics, and then make a preliminary conclusion to the possible relationship between heavy metal concentrations and soil properties and fertilizer inputs types. Univariate analysis of variance was used to determine the significant differences of heavy metal concentrations in the irrigation water. Multivariate analysis of variance (MANOVA) was used to identify the exact sources and demonstrate the significant differences of heavy metal concentrations in soil and crops (wheat and corn grain) resulting from soil parent material, the irrigation water, fertilizer or their interactive effects.

## 3. Result and Discussion

### 3.1. Heavy Metal Concentrations in Soils

Data were evaluated by comparing heavy metals in surface soils of Yucheng City (Table 2) with soil background values of the whole of China, and the lower Yellow River, and the National Environmental quality standards (Table 3). Background heavy metal soil concentrations of the lower Yellow River were calculated by averaging values from 182 surface soil (0–20 cm) samples and 38 subsoil (20–40 cm) samples originally collected in the 1980s [22,23]. As is reported [22,23], for heavy metal concentrations in surface soils (0–20 cm) were significantly higher than those in the subsoil (20–40 cm) of cultivated soil, heavy metal concentrations in the subsoil were chosen to calculate the background levels; otherwise, heavy metal concentrations in surface soils were chosen. For uncultivated soils, heavy metal concentrations in the humus layer were used to calculate the background levels. Background heavy metal soil concentrations were averaged out from the total soil heavy metal concentrations as mentioned above, and it is acceptable if the relative error was below 20% as determined by the samples numbers. The relative errors for Cr, Ni, Cu, Zn, As, Hg, Cd, Pb background value in soil were 2.1%, 3.1%, 3.8%, 2.6%, 8.0%, 8.6%, 3.5%, 3.5%, respectively. So it is credible to use background heavy metal soil concentrations of the lower Yellow River as reference.

Median, mean, minimum (Min) and maximum (Max) heavy metal and soil fertility concentrations of the surface soil sampled in Yucheng City are reported in Table 2. Soil Hg concentrations was skewed through 1-sample K-S test, so median value was used for Hg. The pH of surface soils is between 7.52 and 9.14 (average 8.49). The soils are alkaline (97.7% of samples have pH > 8.0). It is reported that the pH is an essential factor that influences the cation mobility and regulates the solubility of heavy metals in soil [24]. Most of the metals tend to be available in acid pH. The higher soil pH is not favorable for the transference of heavy metals from soil to crops [8]. Cation Exchange Capacity (CEC) refers to the preservation and supply of soil fertilizer and buffering capacity. The mean of soil CEC of Yucheng City is 133.88 mmol·kg^−1^. The range is between 43.05 and 250.43 mmol·kg^−1^, implying that soil in Yucheng City had the moderate capacity to retain and supply fertility [25]. Soil in Yucheng City showed low soil organic matter (SOM) (average 18.96 g·kg^−1^), though a comparison of the 1980 and 2003 levels indicates that SOM is being elevated year by year [26]. This may be attributed to the comprehensive treatment of saline-alkali soil in Yucheng City, such as reusing straw and crop stalks and more organic fertilizer [27,28]. However, organic matter has been found to influence heavy metal absorption in soils due to the CEC of organic material [29]. The low SOM content is not favorable for heavy metal sorption in soils [29]. As for the total P applied to the soil, the available P (avail. P) is much lower, indicating the relatively low P utilization. Heavy metals in soil samples were all significantly higher than background heavy metal soil concentrations of the lower Yellow River except As (ANOVA, F*_pr_* < 0.001). This indicates heavy metal contamination in the agricultural soils of Yucheng City due to long-term agricultural activities. However, we did not see elevated soil average heavy metal concentrations over the National Environmental quality standards, though Ni, Cu, Zn, As, Hg, and Cd concentrations seemed higher than the National Grade One standards. This implies that heavy metals in soil of Yucheng City are at a safe level on the whole due to the high soil pH and low SOM.

### 3.2. Relationship of Soil Heavy Metal Concentration and Soil Fertility Parameters

The concentrations of heavy metals in soil and their impact on ecosystems can be influenced by many factors, such as parent material, climate and anthropogenic activities [30]. Heavy metals may be added to soils in agricultural fertilizers and pesticides, soil amendments(e.g., lime and gypsum), or organic fertilizers (e.g., manures and composts) [31]. Correlation analysis between the soil heavy metals and fertility parameters will help to trace the origins of elevated levels of heavy metals in soil.

In the 86 soils sampled (Table 4), all heavy metal concentrations except Hg showed a significant correlation with soil CEC, Al, and Fe concentrations. These correlations imply either soil contamination has not occurred due to agricultural activities and therefore these elements are associated with indigenous clay minerals, or that some or all heavy metal contamination has been associated with soil types that are rich in Al and Fe. Soil Hg was not correlated with any soil fertility parameter, suggesting diffuse pollution by aerial deposition. However, soil As, Hg, Cd, and Pb were highly correlated with the total P in soil. This may also provide evidences that phosphorus fertilizer use adds heavy metals to the soil. In the Huang-Huai-Hai Plain, 94% of the arable land is in phosphorus deficiency (available P < 10 mg·kg^−1^), and 67% is in severe phosphorus deficiency (available P < 5 mg·kg^−1^) [28]. So large amounts of phosphorus fertilizer inputs have been the primary means to gain higher yields in agriculture [6]. Excessive and inappropriate use of phosphorus fertilizer would inevitably lead to chemical and physical changes in soil.

**Figure 2.**
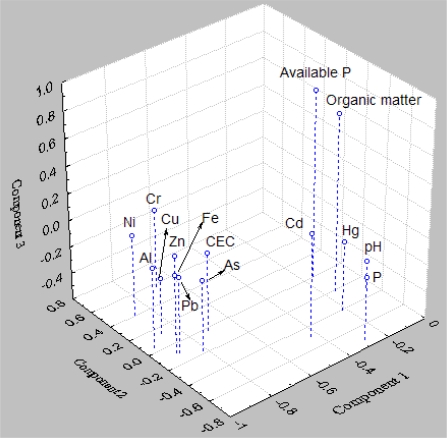
PCA of the heavy metal and fertility parameters for the soils sampled in Yucheng City.

The PCA (Figure 2) showed that the first three principal components of the 15 assessed accounted for 66.77% of the overall variability in the data (Component 1–46.47%; Component 2–10.91%; Component 3–9.40%). Cr, Ni, Cu, Zn, As, and Pb carries almost the same information as Al, Fe, and CEC and have higher loadings on Component 1. Hg and Cd carry information similar to P, and have higher loading on Component 2. These may lend weight to the argument that soil Cd contamination may mostly come from the use of chemical P fertilizer and soil Cr, Ni, As, Hg, Pb, Cu, and Zn have multiple sources such as parent material, organic matter, iron (Fe) and aluminum (Al) oxides, or agricultural activities such as irrigation or fertilizer use. Available P, pH and organic matter seemed to have higher loading on Component 3. This demonstrates that the alkaline soil and low organic matter content go against with heavy metal sorption in soil in Yucheng City.

### 3.3. Heavy Metal Concentrations in Crops

Concentrations of heavy metals in wheat and corn grain varied very much, so median value of heavy metal concentrations was presented in Table 5. The Bioconcentration Factor (BCF) and the Maximum Permitted Concentration (MPC) as recommended by the Ministry of Health of the People’s Republic of China for Cr, Cu, Zn, As, Hg, Cd, and Pb, and FAO/WHO Codex Alimentarius Commission for Ni were also exhibited in Table 5. BCF is a common parameter often used in the study of environmental contamination [32,33]. BCF is the ratio of concentrations of heavy metals in wheat grain and soil.

Cu and Zn were the most abundant metals in wheat and corn grains, as they were the essential micronutrients for plants [34,35]. The high concentration of Zn in wheat and corn grains was related to high concentrations in soil. The BCF value of Zn 0.349 for wheat and 0.237 for corn. The higher BCF of Cu in wheat (0.163) and corn (0.072) reflected the higher Cu concentrations in wheat and corn grain. The results were consistent with prior research on Cu and Zn in wheat [32].

Though Cr was also abundant in soil, Cr in wheat and corn was much lower due to their lower mobility as is shown by their lower BCF values. The results strongly agree with the observation of [32,36] who found that Cr is less mobile in wheat and corn. Still, Ni concentrations of four wheat and two corn grain samples exceeded the MPC level due to the high Ni concentrations in the soil. It is the same with Pb concentrations of three wheat and one corn grain sample.

Cd is usually considered a highly mobile heavy metal in regard to moving from soil to plant. So Cd concentration of one wheat grain sample exceeded the MPC though that of soil was still lower. It is interesting to find that As in wheat grain showed higher concentrations than that as reported [32]. This may result from the soluble As in soil as reported [32]. There are eleven wheat grain samples and three corn grain samples exceeding the MPC.

The BCF of Hg was 0.328 in wheat and 0.046 in corn, which ranked second in wheat and third in corn. It is notable that Hg is not easily transferred from soil to plant, but plants absorb Hg not only from soil but also from aerosols [29], so the aerial Hg may be responsible for the elevated Hg concentrations in grains. The result in 31 wheat grain samples and one corn grain sample reached or exceeded the MPC.

Cr, Ni, Cu, Zn, As, Cd and Pb in wheat and corn may mainly come from the soil in which they were grown. However, aerial Hg may also be the main source for Hg in wheat and corn. The difference of As concentrations between wheat and corn lies in the selective adsorption of heavy metal from soil [29]. It suggests that we should pay attention to health risk from heavy metals derived from agricultural activities in soil and food of Yucheng city.

### 3.4. Soil and Crop Concentrations of Different Agricultural Activities

In order to identify the sources of heavy metal (Cr, Ni, Cu, Zn, As, Hg, Cd, and Pb) levels of different heavy metals in soils and crops (wheat and corn grain) irrigated with water from different sources and fertilized with different manure were assessed separately.

Local rivers, ground water, and the Yellow River are the major sources of the agricultural irrigation in Yucheng City. Heavy metal concentrations in the irrigation water of Yucheng City are shown in Table 6. Compared with the MPC of heavy metals in the Grade 1 standard for surface water recommended by the Ministry of Health, the water for agricultural irrigation in Yucheng City still contained little contamination. Hg concentrations in the local rivers and the Yellow River are significantly lower than in the ground water. Cr concentrations in the Yellow River are significantly higher than in the ground water. Ni and Cu concentrations in the local rivers and the Yellow River are significantly lower than in the ground water. Cr, Pb in the ground water is significantly higher than in the local rivers. There are no significant differences between the local rivers, the Yellow River, and the ground water for Zn, As and Cd. Heavy metals in the local rivers did not show significant differences from those in the Yellow River.

**Figure 3.**
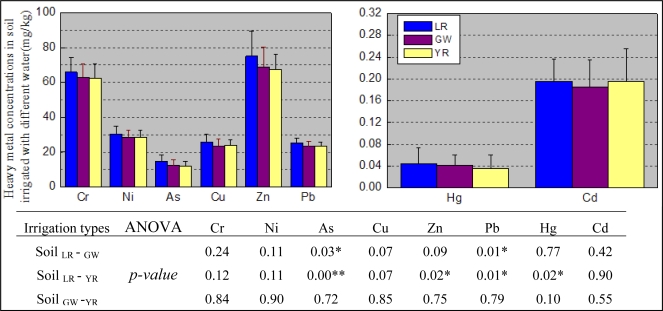
Comparison of heavy metal concentrations in soil irrigated with water from local rivers, ground water and the Yellow River and the ANOVA results (*p-value*). Data represent the mean ± STD. Soil _LR – GW_ refers to the difference between soil irrigated with water from local rivers and groundwater. Soil _LR – YR_ refers to the difference between soil irrigated with water from local rivers and the Yellow River. Soil _GW – YR_ refers to the difference between soil irrigated with water from groundwater and the Yellow River. The same denotation applied to Figure 4.

**Figure 4.**
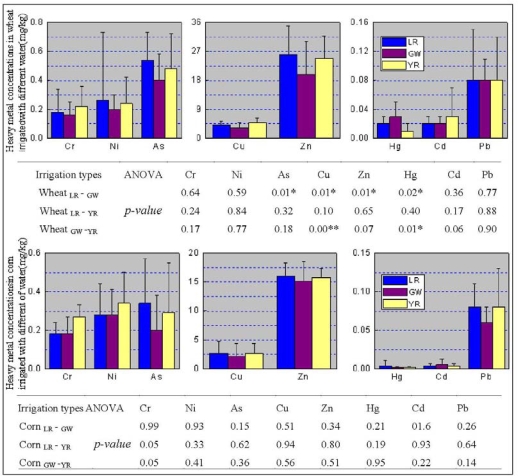
Heavy metal concentrations in wheat and corn derived from different irrigation. Data represent the mean ± STD.

The heavy metal concentrations in soil, wheat and corn grain are shown in Figures 3 and 4. Except for Cd, heavy metal concentrations in soil seemed to follow a sequence. Samples irrigated with water from the local river were greater than those from the ground water which in turn were greater than those from the Yellow River. However, the difference between As and Pb concentrations in soil irrigated with water from local river and the groundwater is statistically significant, and so was the difference between As, Zn, Pb, and Hg concentrations in soil irrigated with water from the local river and the Yellow River. These results may be probative for the argument that long-term irrigation may lead to significant differences in soil heavy metal concentrations. They may also support the argument that multiple sources from agricultural activities are responsible for the significant differences [37]. The inconsistencies between heavy metal concentrations in soil and irrigation water concentrations may result from the numerous sources for soil heavy metals. The differences between As, Cu, Zn and Hg concentrations in wheat grain irrigated with the local rivers and ground water were statistically significant, and so were the differences between Cu and Hg concentrations in wheat grain irrigated with the ground water and the Yellow River (Figure 4). However, no significant differences of heavy metals in corn irrigated with the different sources of irrigation water were found. This may result from the low BCF of corn compared with wheat especially for Ni, Cu, Zn, As, Hg and Cd (Table 5)

**Figure 5.**
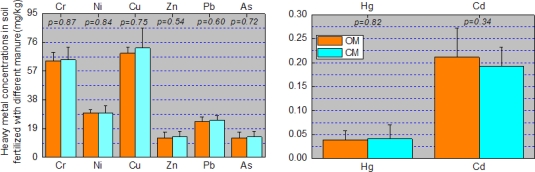
Comparison of heavy metal concentrations in soil fertilized with chemical manure and organic manure and the ANOVA results (*p-value*). Data represent the mean ± STD. CM refers to the chemical manure; OM refers to the organic manure. The same denotation applied to Figures 6.

**Figure 6.**
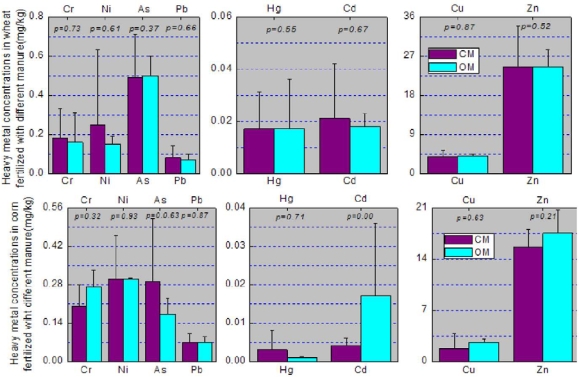
Comparison of heavy metal concentrations in wheat and corn fertilized with chemical manure and organic manure and the ANOVA results (*p-value*). Data represent the mean ± STD.

Heavy metal concentrations in soil, wheat, and corn are shown in Figures 5 and 6 according to the different fertilizers used. From Figure 5 we find there were no significant differences between heavy metals in soil fertilized with chemical manure and organic manure. It is also interesting to find that there were no significant differences between heavy metals in wheat and corn fertilized with chemical manure and organic manure except for Cd in corn (Figure 6). The insignificant differences of heavy metals in soil may be responsible for those in crops, however the exception of Cd in corn may result from the difference of soil Cd and the growth characteristics of corn.

**Figure 7.**
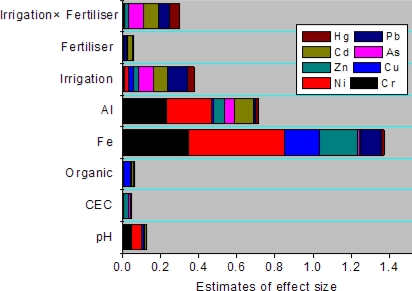
Estimates of effect size (*eta-squared, η^2^*) calculated from the MANOVA for heavy metals in soil.

We chose irrigation water types and fertilizer types as the independent variables, and soil chemical properties (pH, CEC, SOM, Al, Fe) as covariates in the MANOVA to discuss the exact sources of heavy metals in soil based on the prior analysis. Estimate of effect sizes(*eta-squared, η^2^*) describes the ratio of variance explained in the dependent variable by a predictor while controlling for other predictors within the context of MANOVA [38]. Figure 7 showed the *η^2^* values output from MANOVA for soil heavy metal contents. It is easy to find that pH, SOM, CEC contributed least to all heavy metals in soil (ANOVA, *p* > 0.05), which is consistent with the results of PCA. Al, Fe contributed most to soil Cr, Ni (ANOVA, *p* < 0.01). It is reported that generally, anthropic inputs of Cr, Ni in fertilizers, limestone, manure and irrigation water are lower than the concentrations already present in the soil [39]. This indicates a lithogenic control over the distribution of Cr, Ni. It responds to the insignificant differences for Cr, Ni in soil from different agricultural activities (Figures 3–6). Little contribution to soil heavy metal contents from fertilizer use (Figure 7) may account for the insignificant differences between heavy metals in soils and crops fertilized with chemical manure and organic manure. However, the interactive effects of irrigation and fertilizer used contributed a lot to soil As, Cd, Hg, Pb contents (ANOVA, *p* < 0.05) (Figure 7), and lead to the elevated As, Cd, Hg, Pb concentration in wheat and Cd in corn over the MPC. This may support the argument that multiple sources from agricultural activities are responsible for the significant differences [37].

## 4. Conclusions

We used the 1980s heavy metal concentrations in the surface soil of the lower Yellow River as the soil background level of Yucheng City. Heavy metals in soil samples were all significantly higher than the background level except for As (ANOVA, F*_pr_* < 0.001). This indicates heavy metal contamination of the agricultural soils of Yucheng City due to long-term agricultural activities.

The water for agricultural irrigation in Yucheng City contained little contamination. The inconsistency in soil heavy metal concentrations with irrigation water concentrations may result from the numerous sources of soil heavy metals and less influence from irrigation water. The differences between As and Pb concentrations in soil irrigated with water from local river and the ground water were statistically significant, and so were the differences between As, Zn, Pb, and Hg concentrations in soil irrigated with water from the local river and the Yellow River. The differences between As, Cu, Zn and Hg concentrations in wheat grain irrigated with the local rivers and ground water were statistically significant, and so were the differences between Cu and Hg concentrations in wheat grain irrigated with the ground water and the Yellow River. However, no significant differences of heavy metals in corn irrigated with the different sources of irrigation water were found. This may result from the low BCF of corn compared with wheat especially for Ni, Cu, Zn, As, Hg and Cd. There were no significant differences between heavy metals in soil fertilized with chemical manure and organic manure. It is also interesting to find that there were no significant differences between heavy metals in wheat and corn fertilized with chemical manure and organic manure except for Cd in corn. The insignificant differences of heavy metals in soil may be responsible for those in crops, however the exception of Cd in corn may result from the differences of soil Cd and the growth characteristics of corn.

We confirmed that the exact sources of heavy metals in soils and crops through multivariate analysis. Cr, Ni were mainly from the indigenous clay materials. Little contribution to soil heavy metal contents from fertilizer use and the interactive effects of irrigation and fertilization contributed a lot to soil As, Cd, Hg, Pb contents, and lead to the elevated As, Cd, Hg, Pb concentration in wheat and Cd in corn over the MPC.

In sum, there is no obvious contamination of heavy metals in soil and irrigation water. But the long-term accumulation of heavy metals in soil has lead to the local anomalies of heavy metals (As, Cd, Hg, Pb) in wheat and corn. Aerial Hg, however may also be the source of Hg for soil, wheat and corn. As for the numerous sources of soil heavy metals and the unobvious contamination of irrigation water, there is no significant relation between soil heavy metal concentrations and irrigation water concentrations. This study should be helpful for ensuring ecological safety and managing the agricultural development of Yucheng city, the Huang-Huai-Hai Plain, and China in general.

## Figures and Tables

**Table 1.** Analytical results obtained on certified reference materials for soil and crops.

**Elements**	**Observations**	**GSS-5 ± STD**	**Certified ± STD**	**Recovery %**	**GSS-8 ± STD**	**Certified ± STD**	**Recovery %**
Cr (mg/kg)	6	118 ± 10	118.17 ± 8.18	100.14%	68.0 ± 8.0	66 ± 2.7	97.06%
Ni (mg/kg)	6	40 ± 5	40.67 ± 3.33	101.68%	31.5 ± 2.7	33.2 ± 1.9	105.40%
Cu (mg/kg)	6	166 ± 9	164.00 ± 7.32	98.80%	24.3 ± 1.8	23.4 ± 2.2	96.30%
Zn (mg/kg)	6	494 ± 39	491.50 ± 22.13	99.49%	68 ± 6	65.3 ± 2.5	96.03%
As (%)	6	4.12 ± 2.4	414.67 ± 20.55	100.65%	12.7 ± 1.7	12.4 ± 0.5	97.64%
Hg (mg/kg)	6	290 ± 40	302.33 ± 9.48	104.25%	17 ± 4	15.7 ± 3.4	92.35%
Cd (%)	6	045 ± 0.09	0.46 ± 0.05	102.22%	1300 ± 500	1291 ± 70	99.31%
Pb (mg/kg)	6	552 ± 44	562.00 ± 42.98	101.81%	21.0 ± 3.0	21.5 ± 2.3	102.38%

**Elements mg/kg**	**Observations**	**GBW08503 /GSV-3 ± STD**	**Certified ± STD**	**Recovery %**	**GBW10012 /GSV-3 ± STD**	**Certified ± STD**	**Recovery %**

Cr	12	0.55 ± 0.07	0.53 ± 0.04	96.36%	0.55 ± 0.07	0.50 ± 0.01	90.91%
Ni	12	1.9 ± 0.3	2.0 ± 0.1	105.26%	0.097 ± 0.014	0.095 ± 0.014	97.94%
Cu	12	4.40 ± 0.31	4.55 ± 0.41	103.41%	0.66 ± 0.08	0.63 ± 0.08	95.45%
Zn	12	22.7 ± 2.0	21.64 ± 1.44	95.33%	2.9 ± 0.3	3.02 ± 0.42	104.14%
As	12	0.22 ± 0.02	0.21 ± 0.02	95.45%	0.028 ± 0.006	0.03 ± 0.01	107.14%
Hg	12	0.026 ± 0.003	0.025 ± 0.003	96.15%	0.026 ± 0.003	0.025 ± 0.003	96.15%
Cd	12	0.031 ± 0.002	0.031 ± 0.005	100.00%	0.32 ± 0.07	0.33 ± 0.04	103.13%
Pb	12	0.35 ± 0.08	0.36 ± 0.07	102.86%	0.07 ± 0.02	0.07 ± 0.01	100.00%

**Table 2.** Surveyed soil heavy metal concentrations in Yucheng City soils.

Soil	pH	CEC (mmol·kg^−1^)	SOM (g·kg^−1^)	mg·kg^−1^									
				Avail. P	P	Cr	Ni	Cu	Zn	As	Hg	Cd	Pb
Median	8.50	128.89	19.32	11.95	1190.00	63.96	29.37	24.56	69.79	12.83	0.03	0.20	24.29
Mean	8.49	133.88	18.96	12.75	1229.83	64.41	29.46	24.78	71.94	13.38	0.04	0.19	24.37
Min	7.52	43.05	6.54	5.49	767.14	47.76	21.18	15.87	48.49	6.87	0.01	0.10	17.90
Max	9.14	250.43	41.47	25.92	1944.00	89.23	41.18	38.91	124.30	23.20	0.19	0.38	32.28

**Table 3.** Background values of soil heavy metal in the lower Yellow River and the National Environmental quality standards for soils (mg·kg^−1^).

Soil Background values	Cr	Ni	Cu	Zn	As	Hg	Cd	Pb
Lower Yellow River soil	53.60	24.90	21.40	65.10	12.90	0.02	0.09	14.40
Grade One Standard	90.00	40.00	35.00	100.00	15.00	0.15	0.20	35.00
Grade Two Standard	250.00	60.00	100.00	300.00	25.00	1.00	0.60	350.00

**Table 4.** Soil heavy metal concentrations in relation to soil fertility parameters.

	CEC	Avail. P	OM	Cr	Ni	Cu	Zn	As	Hg	Cd	Pb	P	Fe	Al
CEC	1.00													
Avail. P	0.06	1.00												
OM	0.16	0.03**	1.00											
Cr	0.44**	0.11	0.04	1.00										
Ni	0.57**	0.15	0.08	0.87**	1.00									
Cu	0.54**	0.02	0.00	0.65**	0.81**	1.00								
Zn	0.60**	0.12	0.12	0.65**	0.74**	0.71**	1.00							
As	0.55**	0.05	0.08	0.48**	0.62**	0.63**	0.58**	1.00						
Hg	0.12	0.03	0.08	−0.03	0.05	0.15	0.35**	0.17	1.00					
Cd	0.43**	0.17	0.24*	0.11	0.32**	0.44**	0.49**	0.59**	0.13	1.00				
Pb	0.55**	0.14	0.11	0.54**	0.77**	0.80**	0.65**	0.710**	0.29**	0.56**	1.00			
P	0.15	−0.02	0.05	−0.08	−0.02	0.18	0.15	0.38**	0.22*	0.40**	0.33**	1.00		
Fe	0.67**	0.15	0.11	0.60**	0.84**	0.81**	0.74**	0.75**	0.07	0.59**	0.83**	0.21	1.00	
Al	0.62**	0.10	0.11	0.47**	0.73**	0.76**	0.66**	0.76**	0.10	0.63**	0.81**	0.30**	0.96**	1.00

*, ** correlation is significant at the 0.05 and 0.01 levels, respectively (2-tailed).

**Table 5.** Heavy metal distribution (mg·kg^−1^) in crops of Yucheng City, China.

**Plant**	**Cr**	**Ni**	**Cu**	**Zn**	**As**	**Hg**	**Cd**	**Pb**
Wheat(N = 92)								
Median	0.12	0.16	4.06	24.16	0.51	0.01	0.02	0.06
min	0.08	0.03	1.52	5.90	0.08	0.00	0.01	0.03
max	0.85	3.46	9.58	49.16	1.26	0.09	0.20	0.53
BCF(median)	0.002	0.01	0.16	0.35	0.04	0.33	0.10	0.002
Corn(N = 37)								
Median	0.21	0.27	1.68	15.55	0.22	0.002	0.003	0.06
min	0.03	0.11	0.81	6.94	0.03	0.00	0.001	0.03
max	0.37	0.73	8.68	19.79	0.98	0.03	0.03	0.22
BCF(median)	0.003	0.01	0.07	0.24	0.02	0.05	0.02	0.003

MPC	1	0.6	10	50	0.7	0.02	0.1	0.2

**Table 6.** Heavy metal concentrations in the irrigated water of Yucheng City.

**Results**	**Irrigation types (sample)**	**Elements (mg·kg^−1^)**								
			**Cr**	**Ni**	**Cu**	**Zn**	**As**	**Hg**	**Cd**	**Pb**
Statistical results	Local rivers (37)	Mean	3.8	6.26	7.35	10.88	11.57	0.25	0.03	0.67
		Std.	2.32	6.82	4.42	7.69	5.83	0.5	0.02	0.38
	The Yellow River (39)	Mean	13.83	1.49	3.02	7.26	11.74	0.53	0.04	0.18
		Std.	7.65	1.44	3.08	14.89	18.09	0.39	0.04	0.3
	Groundwater (9)	Mean	5.11	6.83	8.36	13.64	17.25	0.13	0.08	2.09
		Std.	3.37	4.69	5.05	11.71	24.51	0.15	0.07	2.79
	Total(85)	Mean	8.54	4.13	5.47	9.51	12.25	0.37	0.04	0.59
		Std.	7.35	5.39	4.51	11.99	14.93	0.45	0.04	1.08

MPC for the surface water^a^			10^b^	-	10	50	50	0.1^c^	1	10

ANVOA results	local rivers-groundwater	F_pr_	0.00**	0.00**	0.00**	0.19	0.96	0.01*	0.38	0.00**
	local rivers-the Yellow River	F_pr_	0.17	0.82	0.55	0.52	0.51	0.49	0.08	0.17
	the Yellow River - groundwater	F_pr_	0.00**	0.01*	0.01*	0.24	0.45	0.00**	0.13	0.07

a refers to the Maximum Permitted Concentration of heavy metals in the Grade 1 standard for surface water (GB3838-2002),

b refers to the Maximum Permitted Concentration of Cr(III) in the Grade 1 standard for surface water (GB3838-2002),

c refers to the Maximum Permitted Concentration of Hg in the Grade 3 standard for surface water (GB3838-2002).

*, ** difference is significant at the 0.05 level and 0.01 levels, respectively (2-tailed); The same denotation applied to Figures 3–6.
